# Psychological treatments for early psychosis can be beneficial or harmful, depending on the therapeutic alliance: an instrumental variable analysis

**DOI:** 10.1017/S003329171500032X

**Published:** 2015-03-25

**Authors:** L. P. Goldsmith, S. W. Lewis, G. Dunn, R. P. Bentall

**Affiliations:** 1 University of Manchester, Manchester, UK; 2 Institute of Brain Behaviour and Mental Health, Institute of Population Health, University of Manchester; Manchester Mental Health and Social Care Trust, Manchester, UK; 3 Department of Psychological Sciences, Liverpool University, Liverpool, UK

**Keywords:** Cognitive therapy, counselling, first episode, psychosis, therapeutic alliance

## Abstract

**Background:**

The quality of the therapeutic alliance (TA) has been invoked to explain the equal effectiveness of different psychotherapies, but prior research is correlational, and does not address the possibility that individuals who form good alliances may have good outcomes without therapy.

**Method:**

We evaluated the causal effect of TA using instrumental variable (structural equation) modelling on data from a three-arm, randomized controlled trial of 308 people in an acute first or second episode of a non-affective psychosis. The trial compared cognitive behavioural therapy (CBT) over 6 weeks plus routine care (RC) *v.* supportive counselling (SC) plus RC *v.* RC alone. We examined the effect of TA, as measured by the client-rated CALPAS, on the primary trial 18-month outcome of symptom severity (PANSS), which was assessed blind to treatment allocation.

**Results:**

Both adjunctive CBT and SC improved 18-month outcomes, compared to RC. We showed that, for both psychological treatments, improving TA improves symptomatic outcome. With a good TA, attending more sessions causes a significantly better outcome on PANSS total score [effect size −2.91, 95% confidence interval (CI) −0.90 to −4.91]. With a poor TA, attending more sessions is detrimental (effect size +7.74, 95% CI +1.03 to +14.45).

**Conclusions:**

This is the first ever demonstration that TA has a *causal* effect on symptomatic outcome of a psychological treatment, and that poor TA is actively detrimental. These effects may extend to other therapeutic modalities and disorders.

## Introduction

Before the 1990s, psychological therapies for people with psychosis and schizophrenia were widely held to be ineffective and potentially harmful. Since then, meta-analyses of the many randomized controlled trials have indicated that cognitive behavioural therapy (CBT) delivered in addition to routine care (RC) is more effective in improving symptoms than RC alone (Tarrier & Wykes, 2004; Zimmermann *et al.* 2005; Wykes *et al.* 2008). However, these trials often report positive results for non-specific psychological therapies (counselling, befriending) used as controls, which also turn out to be better than RC alone (Sensky *et al.* 2000; Tarrier *et al.* 2000).

Randomized controlled trials comparing psychological therapies often either fail to demonstrate a significant difference between treated groups or only demonstrate a small difference, leading to the claim that all therapies are effective but none are more effective than others, sometimes referred to as the ‘Dodo bird conjecture’ (after a character in *Alice Through the Looking Glass*; Wampold, 2001). For example, in an influential meta-analysis of seven common psychological treatments for mild to moderate depression, Cuijpers *et al.* (2008) found no difference in the efficacy of CBT, behavioural activation treatment, psychodynamic treatment, problem-solving therapy or social-skills training. A small increase in efficacy was found for interpersonal psychotherapy (*d* = 0.20), and non-directive supportive treatment was less efficacious than the other therapies (*d* = −0.13). Similarly, in a meta-analysis and meta-regression of brief CBT, counselling or problem-solving therapy for depression or anxiety in primary care, Cape *et al.* (2010) found that, when diagnosis was controlled for, there was no difference in the effectiveness of the three types of therapy. In meta-analyses comparing CBT to other psychosocial treatments for schizophrenia, Jones *et al.* (2012) found no significant differences between psychosocial interventions on positive or negative symptoms, relapse, re-hospitalization, or global mental state measures. Turner & van der Gaag (2014) similarly reported that for overall symptoms, after sensitivity analyses, there were no significant differences between the efficacies of CBT, social skills training and cognitive remediation. Only the comparison between CBT and befriending demonstrated a significant difference for overall symptoms. Where small differences in the efficacy could be detected, the pattern of these differences was consistent with specific foci of specific interventions, for example CBT, was significantly more efficacious than supportive counselling (SC) for reducing positive symptoms, but not for reducing overall symptoms.

Hence, although it is clear that psychosocial treatments are effective for psychosis, it is also clear that the bulk of the effect size is common to all psychosocial interventions. The most parsimonious explanation for this finding is that the major causal mechanism for change is common to all therapies. Identifying the components of therapy with a causal effect is a growing and clinically important area of research, which has recently benefitted from more methodologically sophisticated statistical techniques. This type of research has important implications for the development of more effective interventions, for clinical practice, and for the theoretical understanding of the process underlying therapeutic change (Green & Dunn, 2008).

The non-specific factor most commonly claimed to have a causal effect on outcome is the therapeutic alliance (TA), defined as the quality of the relationship between therapist and client, characterized by trust and a sense of common purpose (Wampold, 2001). For example, in a comparison of interpersonal therapy, CBT, imipramine with clinical management, and placebo with clinical management in the treatment of depression, the patient-reported alliance was reported to predict outcome in both psychotherapies, pharmacotherapy and placebo therapy (Krupnick *et al.* 1996). Meta-analytical reviews of research with mostly non-psychotic patients, across a range of therapies and diagnoses, consistently claim a good alliance predicts positive outcome across different therapy modalities and different conditions, with moderate effect sizes (Martin *et al.* 2000; Horvath & Symonds, 2001; McLeod, 2011). However, the question of real interest is whether a good TA causes a better outcome, rather than merely correlates with it, in the sense that patients set to have a good outcome regardless of therapy might be those in whom a good TA occurs. All prior research has used a simple ‘ordinary least squares’ regression or correlation analysis and so is not protected from this possibility. This limitation is addressed here by using instrumental variables in a structural equation model, which allows causal interpretations (Pearl, 1998; Halpern & Pearl, 2005).

There is already evidence to suggest a heterogeneous response to therapy in patients with psychosis. Dunn *et al.* (2012) in an analysis of the Psychological Prevention of Relapse in Psychosis (PRP) trial, found that, although receipt of full therapy including specific cognitive and behavioural techniques improved clinical outcomes, delivery of partial therapy involving engagement and assessment was not effective; indeed the analysis indicated that for patients with low levels of engagement who are not receiving full therapy, persistence in trying to deliver full therapy can have a detrimental effect on symptoms. This was in contrast to the overall intention-to-treat result of the trial which showed no or minimal effect of CBT, since full therapy was only delivered to a minority. The authors suggest that problems in establishing the TA may have been one of the reasons why full therapy was not delivered to more individuals. For this reason, the present research also investigates the effect of therapy at different levels of alliance.

The SoCRATES randomized controlled trial of CBT for acutely ill first and second episode patients (Lewis *et al.* 2002; Tarrier *et al.* 2004) was chosen for the present study as (i) measures of the TA were taken, and (ii) it is of high quality; having been independently assessed as the highest quality of 18 trials of CBT for psychosis in a meta-analytical review (Wykes *et al.* 2008). Strengths of the trial included a large sample (308 patients aged 21–35 years), rigorously blinded assessments, high follow-up rates, multiple assessment points, and checks for assessor reliability. Participants included both inpatients and outpatients and the study received ethical approval from a National Health Service Research Ethics Committee. All participants gave informed consent. There were three arms: CBT, SC (both delivered in addition to RC over 6 weeks in the acute phase with booster sessions) and RC only. RC comprised in most cases inpatient care and antipsychotic medication. Both CBT and SC were manualized and supervised by expert practitioners. Fidelity to CBT (e.g. Socratic questioning style, completion of homework) was independently rated from taped sessions using the psychosis version of the Cognitive Therapy Scale (Haddock *et al.* 2001) and was assessed as high for the CBT arm and low for the SC arm, indicating high-quality therapy. The main trial results are reported elsewhere (Lewis *et al.* 2002; Tarrier *et al.* 2004); this paper reports a secondary analysis. The 18-month follow-up found that both CBT and SC patients fared better on the primary outcome total Positive and Negative Syndrome Scale score (PANSS; Kay *et al.* 1987) than patients receiving RC alone (Tarrier *et al.* 2004) with an effect estimate of −6.22 (s.e. = 2.17, *p* = 0.005).

Using instrumental variable methods, as proposed and explained in detail in a methodological paper by Dunn & Bentall (2007), we aimed to evaluate the causal effect of the TA as a modifier of the dose-effect of number of sessions attended on the effect of treatment and, subsequently, (1) evaluate the effect of the number of sessions attended at the maximum alliance; (2) evaluate the effect of the number of sessions attended at the poorest level of the TA and (3) to estimate the causal effect on outcome if therapy had occurred at one unit better alliance.

## Method

Stata IC/13.1 (StataCorp, 2013) was used for all statistical analyses. The data used in the analyses is obtainable in Stata format from http://www.population-health.manchester.ac.uk/biostatistics/research/data/.

Patients meeting DSM-IV criteria for schizophrenia or schizophreniform disorder during a first (80%) or a second (20%) episode were randomized within 2 weeks to one of three treatment groups. The two psychological treatment groups received weekly sessions for the first 6 weeks, followed by two boosters.

TA was measured using two measures: a therapist-rated measure [the psychotherapy status report (PSR; Frank & Gunderson, 1990), and the self-report version of the California Therapeutic Alliance Scales (CALPAS; Marmar *et al.* 1989)]. The CALPAS was used in this research as self-report measures correlate more strongly with outcome than therapist-rated measures (Horvath & Symonds, 2001). As already noted, there was no significant difference between the improvement in the CBT and SC groups Tarrier *et al.* (2004); the effect estimate for this comparison was −0.85 (s.e. = 2.42, *p* = 0.725). This similarity of outcomes was likely due to factors common to both therapies and cannot be explained by the SC group inadvertently receiving a version of CBT or vice versa as fidelity to CBT was good (see above).

**Fig. 1.**
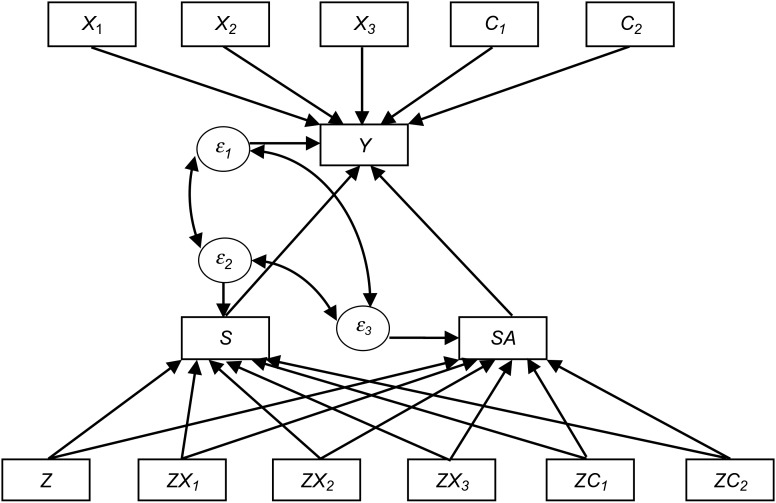
*X*_1_, *X*_2_ and *X*_3_ are baseline variables: the baseline Positive and Negative Syndrome Scale (PANSS) total score, years of education and log of the duration of untreated psychosis. *C*_1_ and *C*_2_ are different centres. The bottom row includes randomization, *Z* (coded in binary) and the interaction of randomization and the baseline variables. Outcome, *Y* is the 18-month PANSS total score. *S* is sessions and *SA* is the interaction of sessions and alliance. Measurement errors are labelled *ε*_1_, *ε*_2_ and *ε*_*3*_. The bottom row therefore shows the interaction of randomization and variables having a causal effect on the number of sessions attended and the interaction of sessions and alliance (the effect of alliance is modelled in a dose-response manner). In turn, these post-randomization variables have a causal effect on the symptomatic outcome (the PANSS 18-month outcome score). The paths connecting the post-randomization variables to outcome are of primary interest and these are shown in the results section as *β*_*S*_ and *β*_*SA*_. The top row of Fig. 1 shows the baseline variables directly affecting the PANSS 18-month score. The strength of these causal relationships is the same for patients randomized to receive a psychological therapy or not. By using the interactions of randomization and baseline variables in the bottom row, and coding randomization to treatment as usual only as 0, patients not receiving a talking therapy are not included in the causal pathway estimates in the bottom part of the diagram.

Alliance measures from the fourth session were used, as this is late enough in the therapy for the alliance to have developed, but not too late for it to be an indirect measure of outcome. The CALPAS scores range from 0 to 7 (calculated as the mean items score). In the treated group, the 4th session CALPAS score had 45% missing data, and the number of sessions attended had 12% missing data. The primary outcome measure was the total score on the PANSS, administered by three research psychiatrists blinded to treatment allocation at 18-month follow-up. Structural equation modelling with full information maximum likelihood (SEM FIML) was used to fit the model. The structural equation model is shown in Fig. 1. The parameters of the SEM model were evaluated simultaneously at the point at which they cross the axes (i.e. the point at which the variable is equal to zero), as is customary. Observing the figure, the parameter for sessions (*S*) gives the effect of an additional session when alliance is zero and the parameter for the interaction of sessions and alliance (*SA*) gives the effect of increasing the alliance from zero to one. The interaction of sessions and alliance was used in the absence of a main effect alliance alone, as alliance can only have a moderating effect on the effects of the number of sessions attended, and cannot influence outcome (and is not defined) in the absence of therapy. The parameters for *S* and *SA* are shown in the Results section as *β*_*S*_ and *β*_*SA*_ respectively. The model allows for the possibility that additional sessions may have different effects at different levels of alliance. To explore this possibility the model was fitted twice using first the CALPAS scores as they were originally scaled (with 0 the minimum CALPAS score) and then simply re-scaled (by −7) to make 0 the maximum CALPAS score.

Instrumental variable methods were used to control for the potential effects of additional variables (hidden common causes, i.e. confounders) affecting the relationship between both the number of sessions attended and level of TA on outcome. The instrumental variables are the interactions between randomization and baseline covariates on the bottom line of Fig. 1. They influence outcome but this influence is fully explained by their effects on the number of sessions attended and the product of sessions and the TA. The variables used were selected for these properties. The baseline variables used to create instruments are log DUP (the log of the duration of untreated psychosis), years of education, baseline PANSS score, and centre (a binary variable; three different centres were used; two of which were included explicitly in the model, leaving the other centre implicitly coded as the comparison centre). No further variables were used as models with fewer variables have better statistical properties and are more parsimonious.

The random disturbances (‘errors’) included in the causal model (*ε*_1_, *ε*_2_, *ε*_3_) show that the symptomatic outcome, dose and the interaction of dose and alliance are all modelled as imperfectly predicted. Importantly, allowing for correlation between these errors (shown with curved bidirectional arrows), allows for hidden confounding, the problem that makes most research investigating the effect of post-randomization variables merely *correlational* rather than *causal*. To state this more clearly, not allowing for these correlations between post-randomization variables and outcome would not have excluded the possibility that those who tend to develop good alliance also tend to have a good outcome (i.e. that perhaps the alliance does not cause the good outcome).

Full Information Maximum Likelihood (FIML) estimation was used to fit the instrumental variable model, a procedure that includes cases with missing data, grouping data according to missing data patterns. Probable values for the missing data are implied by the observed values. FIML deals appropriately with data missing at random, and has a superior statistical profile than a complete cases analysis, which only analyses the observed data (referred to as listwise deletion in the SEM literature) (Yuan *et al*. 2012; Enders & Bandalos, 2001).

## Ethical standards

The authors assert that all procedures contributing to this work comply with the ethical standards of the relevant national and institutional committees on human experimentation and with the Helsinki Declaration of 1975, as revised in 2008.

## Results

Firstly, summary statistics from the SoCRATES trial are presented in Table 1. The results of the analysis described in this paper are presented in Table 2. The core (causal) parameters of our statistical model are the effects of increasing sessions (when alliance is coded to be zero: *β*_*S*_) and of increasing the product of sessions and alliance (the interaction: *β*_*SA*_) on outcome (18-month PANSS score). The strength of the TA (CALPAS score) is coded in two ways: (*a*) from −7 (lowest alliance) to 0 (maximum alliance), and (*b*) from 0 (lowest alliance) to +7 (maximum alliance). This makes no difference to the fit of the model (they are mathematically equivalent) but the use of each option aids the interpretation of the parameter *β*_*S*_). For option (*a*) *β*_*S*_ is the effect of sessions at maximum alliance; for option (*b*) *β*_*S*_ is the effect of sessions at minimum alliance. The interaction effect (*β*_*SA*_) should be unaffected. We fit the models to two different data sets: (1) including only those subjects for which there is a measure of both sessions attended and the TA available, and (2) using the full dataset, allowing for the gaps (missing values) in the data.

**Table 1.** Summary statistics from the SoCRATES trial by centre

	Treated groups (CBT and SC combined)			Untreated (RC only) group		
	Mean (s.d.), % missing			Mean (s.d.), % missing		
	Centre 1 (Liverpool) (*N* = 75)	Centre 2 (Manchester) (*N* = 76)	Centre 3 (Nottinghamshire) (*N* = 56)	Centre 1 (Liverpool) (*N* = 38)	Centre 2 (Manchester) (*N* = 36)	Centre 3 (Nottinghamshire) (*N* = 27)
Baseline variables						
Baseline PANSS score	82.33 (15.54), 0	100.51 (15.86), 0	81.93 (11.65), 0	79.97 (12.68), 0	97.47 (16.64), 0	83.70 (15.94), 0
Log DUP	1.15 (0.53), 0	1.41 (0.63), 0	0.82 (0.44), 0	1.05 (0.45), 0	1.40 (0.60), 0	0.81 (0.40), 0
Years of education	11.45 (2.36), 0	11.47 (1.87), 0	11.41 (2.75), 0	11.29 (1.77), 0	12.70 (2.36), 0	11.67 (2.92), 0
Intermediate outcomes						
4th session CALPAS score (*A*)^a^	5.57 (0.86), 48.0	5.05 (0.88), 34.2	5.19 (1.42), 55.4	–	–	–
Dose (*S*)^a^	15.67 (5.94), 24.0	14.00 (6.27), 6.6	11.07 (5.26), 3.6	–	–	–
SA^a^	103.33 (22.14), 60.0	81.15 (28.59), 34.2	70.00 (31.94), 57.1	–	–	–
Outcome variable						
PANSS 18-month total	55.02 (14.59), 37.3	76.52 (20.02), 29.0	53.16 (8.76), 10.7	71.84 (15.22), 50.0	75.50 (22.34), 27.8	56.19 (10.45), 22.2

CBT, Cognitive behavioural therapy; SC, supportive counselling; RC, routine care; PANSS, Positive and Negative Syndrome Scale; DUP, duration of untreated psychosis; CALPAS, California Therapeutic Alliance Scales.

a The missing data for intermediate outcomes refers to the treated group only.

**Table 2.** Causal estimands for the effect of sessions and alliance

	Estimate	s.e.	*p*	95% CI
**(*a*) Evaluated when best alliance = 0**				
(1) SEM ignoring participants without data on both sessions and alliance				
*β*_S_ (effect of sessions at best alliance)	−2.66	0.76	0.000	−4.15 to −1.17
*β*_SA_ (the interaction)	−1.44	0.50	0.004	−2.42 to −0.47
(2) SEM FIML including all participants’ data				
*β*_S_ (effect of sessions at best alliance)	−2.91	1.02	0.005	−4.91 to −0.90
*β*_SA_ (the interaction)	−1.52	0.63	0.016	−2.76 to −0.29
**(*b*) Evaluated when worst alliance = 0**				
(1) SEM ignoring participants without data on both sessions and alliance				
*β*_S_ (effect of sessions at worst alliance)	7.45	2.76	0.007	2.04 to 12.87
*β*_SA_ (the interaction)	−1.45	0.50	0.004	−2.42 to −0.47
(2) SEM FIML including all participants’ data				
*β*_S_ (effect of sessions at worst alliance)	7.74	3.42	0.024	1.03 to 14.45
*β*_SA_ (the interaction)	−1.52	0.63	0.016	−2.76 to −0.29

CI, Confidence interval; SEM FIML, structural equation modelling with full information maximum likelihood.

Given the relatively large proportion of trial participants with a missing TA measure, it is important that the causal parameters of the explanatory models are fitted using methods that satisfactorily allow for these missing data (i.e. using FIML SEM). The initial methodological work of Dunn & Bentall (2007) was illustrated using only those SoCRATES participants who had non-missing data for both sessions and alliance. Here we focus on the FIML SEM estimates. However, our findings do not appear to be unduly influenced by the method chosen to deal with missing data.

The key substantive finding is that there is a statistically significant sessions by alliance interaction effect on outcome. That is, the effect of increasing the number of sessions attended is dependent on the participants’ reported TA. At one extreme (a reported CALPAS score of 7 – the best level of alliance) increasing the number of sessions attended improves the effectiveness of the therapy (total 18-month PANSS score reduced, on average, by just under 3 points for every session attended). At the other extreme (a reported CALPAS score of 0 – the worst level of alliance) increasing the number of sessions attended makes things worse – the therapy appears to be detrimental (total 18-month PANSS score increased, on average, by about 7.5 points for every session attended). As the confidence intervals for each estimate both have the same sign as the estimate (e.g. the confidence intervals for positive effects are both positive), there is a high level of certainty that these effects exist.

## Discussion

Much empirical evidence suggests that the nature and strength of the TA established between therapist and client explains some of the effect of psychotherapies. This appears to be so across different types of therapy, and across diagnostic boundaries. However, until now, it has not been possible to be sure that this is a truly causal relationship: that a good TA contributes to, rather than simply correlates with, the good outcome. This study used structural equation modelling to deal with unmeasured confounding (correlation between post-randomization variables and outcome) and potential bias due to missing data. We have presented three main findings: in psychological therapies for psychosis, (i) a good TA causes improvement; (ii) a poor TA has a detrimental effect, and (iii) improving the alliance causes a better outcome. Hence, the findings provide strong evidence that the TA was a common causal factor contributing to the outcome of patients receiving either of the therapies, CBT or SC. If this finding proves generalizable to other therapies and conditions, it may tend to support for the Dodo bird conjecture that all therapies are (usually) equally effective (Wampold, 2001) and help to explain why this is the case.

The finding that therapy may have a detrimental effect when the alliance is poor is equally important and perhaps an echo of traditional attitudes about treatment for schizophrenia, which dictated that any discussion of the details of the psychosis or delusional ideas should be avoided, as it would exacerbate the psychosis or constitute ‘inadvertent collusion’ (McCabe & Priebe, 2008). Hence, although CBT has been indicated to be effective in many randomized controlled trials, the present findings indicate that therapy should proceed with caution if the TA is poor. A corollary is that CBT is more likely to be an effective treatment for psychosis if only delivered when a good TA is possible. Current debates about the effectiveness of CBT (e.g. Jauhar *et al*. 2014) that fail to address the heterogeneity of treatment response may underestimate the effects of treatment in optimal circumstances.

Previous studies have reported the TA to be a predictor of other important outcomes in severe mental illness, for example attitudes to medication (Day *et al.* 2005), adherence to antipsychotic medication (McCabe *et al.* 2012), the effectiveness of community mental health case management (Howgego *et al.* 2003), maintenance of contact with services (Johansson & Eklund, 2006) and supported workplace performance (Davis & Lysaker, 2007), indicating the importance of the quality of therapeutic relationships in many aspects of recovery from psychosis. The statistical methodology used in this paper extends our understanding, showing that for psychological therapies, the relationship is not just correlational, but causal. Hence, an important clinical implication is that psychiatric services may be able to improve outcomes by improving the quality of therapeutic relationships across the range of interactions, and by developing a more personalized approach in which interventions are tailored to patients’ needs.

The limitations of the present paper are that the variation in alliance was constrained by the model to be all due to client variation, i.e. as the assignment of therapist was not randomized, it was not possible to model the variation in TA due to the therapist, a model which would acknowledge that some therapists tend to form better alliances than others (Wampold, 2001). This issue would benefit from further research. Additionally, further research may investigate whether the causal effect of the TA is observed in therapies for other conditions. It might be argued that another limitation is our inclusion of both the CBT- and SC-treated patients in the same analysis, as the two therapies place a different emphasis on the importance of the alliance as a mechanism, at least in theory. For example, Rogers (1957) perhaps the leading advocate of the SL approach, emphasized the quality of the therapeutic relationship as a necessary and sufficient condition for successful therapy whereas CBT therapists tend to see the alliance as more instrumental in ensuring the patient's adherence to the treatment protocol (e.g. Dunn *et al.* 2006). However, separating the groups or studying only one of the conditions would have severely limited the numbers available, and the two interventions did not produce significantly different outcomes (Tarrier *et al.* 2004). Moreover, the focus of our approach has been to test the hypothesis that the alliance is a common mechanism affecting outcome, as might be expected according to the Dodo bird conjecture (Wampold, 2001).

### Strengths and weaknesses in relation to other studies

A small number of prior studies have examined the relationship between the TA and outcome in psychotic patients receiving psychotherapy. Gunderson *et al*.'s (1984) clinical trial of psychodynamic psychotherapy reported the alliance, as rated by the therapist, strongly predicted outcome even when other variables, for example, symptom severity at inception, were taken into account. Svensson & Hansson (2007) reported a positive relationship between initial patient-rated alliance scores and outcome in 26 schizophrenia-spectrum patients treated with CBT. Similarly, the meta-analytical reviews, which consider mostly non-psychotic patients, across a range of therapies and diagnoses, find that the alliance predicts outcome moderately well (Martin *et al.* 2000; Horvath & Symonds, 2001; McLeod, 2011). However, these studies essentially calculated the correlation between alliance and outcome. The prior research concerning TA can be interpreted as indicating that patients who tend to form good alliances also tend to have a better prognosis, or, at least, it is not protected methodologically from such an interpretation. In terms of inferring causality, these studies are methodologically confounded by essentially just correlating the alliance score with outcome.

This research dealt with that problem by using structural equation modelling, and allowing for correlation between post-randomization variables and outcome separately to the causal effect. Missing data, which can bias estimates or suggest inaccurate confidence intervals, was addressed using FIML estimation.

## Conclusions

This analysis shows clearly the causal effect of TA on the outcome of psychotherapy for psychosis. The results indicate that at high levels of alliance, therapy is beneficial, but that at low levels of alliance, therapy is detrimental. Clinically, this suggests that establishing a good alliance in psychotherapy for psychosis is essential for a patient to benefit from therapy and that if the alliance is poor, persistence in trying to engage the client in psychotherapy is not appropriate. Future trials of psychological treatments for psychosis should consider methods of maximizing the alliance, or at least employ procedures for discontinuing therapy if the alliance is poor. A wider implication is that psychiatric services should prioritize ensuring that all staff engage effectively with patients.

## References

[ref36] Cape J, Whittington C, Buszewicz M, Wallace P, Underwood L (2010). Brief psychological therapies for anxiety and depression in primary care: meta-analysis and meta-regression. BMC Medicine 8, 38–51. 20579335 10.1186/1741-7015-8-38 PMC2908553

[ref37] Cuijpers P, van Straten A, Andersson G, van Oppen P (2008). Psychotherapy for depression in adults: a meta-analysis of comparative outcome studies. Journal of Consulting and Clinical Psychology 76, 909–922. 19045960 10.1037/a0013075

[ref1] Davis LW, Lysaker PH (2007). Therapeutic alliance and improvements in work performance over time in patients with schizophrenia. Journal of Nervous and Mental Disorders 195, 353–357. 10.1097/01.nmd.0000261954.36030.a1 17435487

[ref2] Day JC, Bentall RP, Roberts C, Randall F, Rogers A, Cattell D, Healy D, Rae P, Power C (2005). Attitudes toward antipsychotic medication: the impact of clinical variables and relationships with health professionals. Archives of General Psychiatry 62, 717–724. 15997012 10.1001/archpsyc.62.7.717

[ref3] Dunn G, Bentall R (2007). Modelling treatment-effect heterogeneity in randomized controlled trials of complex interventions (psychological treatments). Statistics in Medicine 26, 4719–4745. 17476649 10.1002/sim.2891

[ref4] Dunn G, Fowler D, Rollinson R, Freeman D, Kuipers E, Smith B, Steel C, Onuwmere J, Jolley S, Garety P, Bebbington P (2012). Effective elements of cognitive behaviour therapy for psychosis: results of a novel type of subgroup analysis based on principal stratification. Psychological Medicine 42, 1057–1068. 21939591 10.1017/S0033291711001954 PMC3315767

[ref5] Dunn H, Morrison AP, Bentall RP (2006). The relationship between patient suitability, therapeutic alliance, homework compliance and outcome in cognitive therapy for psychosis. Clinical Psychology and Psychotherapy, 13, 145–152.

[ref38] Enders CK, Bandalos DL (2001). The relative performance of full information maximum likelihood estimation for missing data in structural equation models. Structural Equation Modelling 8, 430–457.

[ref6] Frank AF, Gunderson JG (1990). The role of therapeutic alliance in the treatment of schizophrenia. Archives of General Psychiatry 47, 228–236. 1968329 10.1001/archpsyc.1990.01810150028006

[ref7] Green J, Dunn G (2008). Using intervention trials in developmental psychiatry to illuminate basic science. British Journal of Psychiatry 192, 323–325. 18450651 10.1192/bjp.bp.107.046284

[ref8] Gunderson JG, Frank AF, Katz HM, Vannicelli ML, Frosch JP, Knapp PH (1984). Effects of psychotherapy in schizophrenia: II. Comparative outcome of two forms of treatment. Schizophrenia Bulletin 10, 564–598. 6151246 10.1093/schbul/10.4.564

[ref9] Haddock G, Devane S, Bradshaw T, McGovern J, Tarrier N, Kinderman P, Baguley I, Lancashire S, Harris N (2001). An investigation into the psychometric properties of the cognitive therapy scale for psychosis (CTS-Psy). Behavioural and Cognitive Psychotherapy 29, 93–106.

[ref10] Halpern JY, Pearl J (2005). Causes and explanations: a structural model approach. Part 1: causes. British Journal for the Philosophy of Science 56, 843–887.

[ref11] Horvath AO, Symonds BD (2001). Relation between working alliance and outcome in psychotherapy: a meta-analysis. Journal of Consulting and Clinical Psychology 38, 139–149.

[ref12] Howgego IM, Yellowlees P, Owen C, Meldrum L, Dark F (2003). The therapeutic alliance: the key to effective patient outcome? A descriptive review of the evidence in community mental health case management. Australian and New Zealand Journal of Psychiatry 37, 169–183. 12656956 10.1046/j.1440-1614.2003.01131.x

[ref13] Jauhar S, McKenna PJ, Radua E, Fung R, Salvador R, Laws KR (2014). Cognitive-behavioural therapy for the symptoms of schizophrenia: systematic review and meta-analysis with examination of potential bias. British Journal of Psychiatry 204, 20–29. 24385461 10.1192/bjp.bp.112.116285

[ref14] Johansson H, Eklund M (2006). Helping alliance and early dropout from psychiatric out-patient care: the influence of patient factors. Social Psychiatry and Psychiatric Epidemiology 41, 140–147. 16372143 10.1007/s00127-005-0009-z

[ref15] Jones C, Hacker D, Cormac I, Meaden A, Irving CB (2012). Cognitive behavioural therapy versus other psychosocial treatments for schizophrenia (Review). Cochrane Database of Systematic Reviews 2012. Art. no.: CD008712. doi: 10.1002/14651858.CD008712.pub2. PMC4163968 22513966

[ref16] Kay SR, Fiszbein A, Opler LA (1987). The Positive and Negative Syndrome Scale (PANSS) for schizophrenia. Schizophrenia Bulletin 13, 507–518. 10.1093/schbul/13.2.261 3616518

[ref17] Krupnick JL, Sotsky SM, Simmens S, Moyer J, Elkin I, Watkins J, Pilkonis PA (1996). The role of the therapeutic alliance in psychotherapy and pharmacotherapy outcome: findings in the National Institute of Mental Health Treatment of Depression Collaborative Research Program. Journal of Consulting and Clinical Psychology 64, 532–539. 8698947 10.1037//0022-006x.64.3.532

[ref18] Lewis SW, Tarrier N, Haddock G, Bentall R, Kinderman P, Kingdon D, Siddle R, Drake R, Everitt K, Leadley K, Benn A, Grazebrook K, Haley C, Akhtar S, Davies L, Palmer S, Faragher B, Dunn G (2002). Randomised controlled trial of cognitive behaviour therapy in early schizophrenia: acute-phase outcomes. British Journal of Psychiatry 181 (Suppl. 43), 91–97. 12271807 10.1192/bjp.181.43.s91

[ref19] Marmar CR, Gaston L, Gallagher D, Thompson LW (1989). Towards the validation of the California Therapeutic Alliance Rating System. Psychological Assessment 1, 46–52.

[ref20] Martin DJ, Garske JP, Davis MK (2000). Relation of the therapeutic alliance with outcome and other variables: a meta-analytic review. Journal of Consulting and Clinical Psychology 68, 438–450. 10883561

[ref21] McCabe R, Bullenkamp J, Hansson L, Lauber C, Martinez-Leal R, Rössler W, Salize HJ, Svensson B, Torres-Gonzalez F, van den Brink R, Wiersama D, Priebe S (2012). The therapeutic relationship and adherence to antipsychotic medication in schizophrenia. PLoS ONE 7, 4, . 10.1371/journal.pone.0036080 PMC3338634 22558336

[ref22] McCabe R, Priebe S (2008). Communication and psychosis: it's good to talk, but how? British Journal of Psychiatry 192, 404–405. 18515888 10.1192/bjp.bp.107.048678

[ref23] McLeod BD (2011). The relation of the alliance with outcomes in youth psychotherapy: a meta-analysis. Clinical Psychology Review 31, 603–616. 21482319 10.1016/j.cpr.2011.02.001

[ref24] Pearl J (1998). Graphs, causality and structural equation models. Sociological Methods and Research 27, 226.

[ref25] Rogers CR (1957). The necessary and sufficient conditions of therapeutic personality change. Journal of Consulting Psychology 21, 95–103. 13416422 10.1037/h0045357

[ref26] Sensky T, Turkington D, Kingdon D, Scott JL, Scot J, Siddle R, O'Carroll M, Barnes TRE (2000). A randomized controlled trial of cognitive-behavioral therapy for persistent symptoms in schizophrenia resistant to medication. Archives of General Psychiatry 57, 165–172. 10665619 10.1001/archpsyc.57.2.165

[ref27] StataCorp (2013). Stata Statistical Software: Release 13. StataCorp LP: College Station, TX.

[ref28] Svensson B, Hansson L (2007). Therapeutic alliance in cognitive therapy for schizophrenic and other long-term mentally ill patients: development and relationship to outcome in an in-patient treatment programme. Acta Psychiatrica Scandinavica 99, 281–287. 10223431 10.1111/j.1600-0447.1999.tb07226.x

[ref29] Tarrier N, Kinney C, McCarthy E, Humphreys L, Wittkowski A, Morris J (2000). Two-year follow-up of cogitve-behavioral therapy and supportive counselling in the treatment of persistent symptoms in chronic schizophrenia. Journal of Consulting and Clinical Psychology 68, 917–922. 11068978

[ref30] Tarrier N, Lewis SW, Haddock G, Bentall R, Drake R, Kinderman P, Kingdon D, Siddle R, Everitt J, Leadley K, Benn A, Grazebrook K, Haley C, Akhtar S, Davies L, Palmer S, Dunn G (2004). Cognitive-behavioural therapy in first-episode and early schizophrenia: 18-month follow-up of a randomised controlled trial. British Journal of Psychiatry 184, 231–239. 14990521 10.1192/bjp.184.3.231

[ref31] Tarrier N, Wykes T (2004). Is there evidence that cognitive behaviour therapy is an effective treatment for schizophrenia? A cautious or cautionary tale? Behaviour Research and Therapy 42, 1377–1401. 15500811 10.1016/j.brat.2004.06.020

[ref32] Turner DT, van der Gaag M (2014). Psychological Interventions for Psychosis: a meta-analysis of comparative outcome studies. American Journal of Psychiatry 171, 523–538. 24525715 10.1176/appi.ajp.2013.13081159

[ref33] Wampold BE (2001). The Great Psychotherapy Debate: Models, Methods and Findings. Lawrence Erlbaum Associates: Mahwah, NJ.

[ref34] Wykes T, Steel C, Everitt B, Tarrier N (2008). Cognitive behavior therapy for schizophrenia: effect sizes, clinical models, and methodological rigor. Schizophrenia Bulletin 34, 523–537. 17962231 10.1093/schbul/sbm114 PMC2632426

[ref39] Yuan K-H, Yang-Wallentin F, Bentler PM (2012). ML versus MI for missing data with violation of distribution conditions. Sociological Methods and Research 41, 598–629. 24764604 10.1177/0049124112460373 PMC3995817

[ref35] Zimmermann G, Favrod J, Trieu VH, Pomini V (2005). The effect of cognitive behavioural treatment on the positive symptoms of schizophrenia spectrum disorders: a meta-analysis. Schizophrenia Research 77, 1–9. 16005380 10.1016/j.schres.2005.02.018

